# Experimental Diabetes

**Published:** 1887-03

**Authors:** 


EXPERIMENTAL DIABETES.—The usual experiments are successful only when the liver contains glycogen. Mering, of Strasburg, finds that a dose of one grain of phloridzin ($C^{21} H^{24} O^{10}$, an extract from root-bark of fruit trees) to every thousand grains of body weight, in the case of dogs, produced ten per cent. of grape sugar in the urine, irrespective of the liver's containing glycogen. By increasing the dose the sugar reached fifteen per cent., and the animals were well and sprightly. The quantity of sugar in the urine depended on the amount of phloridzin, but this dependence ceased to hold good if the animal had food—flesh or bread. Curare in hungered animals caused no glycosuria; but a dog, after fasting three weeks, after a dose of phloridzin, had eight per cent. of sugar in its urine.

Mering rendered the liver functionless by phosphorus poisoning, and sugar persisted in the urine. In geese, after removal of the liver, severe glycosuria persisted. The liver of a dog suffering severe phloridzin glycosuria gave but six grains of glycogen—very little.

The amount of sugar in the blood more than once showed a diminution, although there was ten to fifteen per cent. of sugar in the urine. This differs remarkably from what has been found in other cases of experimental diabetes. For true diabetes, the records vary.

Mering believes that the cause of diabetes is probably an alteration in the *kidneys*. Or one can imagine an alteration in the blood itself, so that the sugar is not retained as in health, but passes into the urine. —*Medical Chronicle*, Manchester, Eng.

